# Benchmarking the clinical outcomes of Healthentia SaMD in chronic disease management: a systematic literature review comparison

**DOI:** 10.3389/fpubh.2024.1488687

**Published:** 2024-12-24

**Authors:** Sofoklis Kyriazakos, Aristodemos Pnevmatikakis, Konstantina Kostopoulou, Laurent Ferrière, Kyun Thibaut, Erika Giacobini, Roberta Pastorino, Marco Gorini, Peter Fenici

**Affiliations:** ^1^Innovation Sprint srl, Clos Chapelle-aux-Champs, Brussels, Belgium; ^2^COVARTIM, Watermael-Boitsfort, Belgium; ^3^Section of Hygiene, Department of Life Sciences and Public Health, Università Cattolica del Sacro Cuore, Rome, Italy; ^4^Department of Woman and Child Health and Public Health, Fondazione Policlinico Universitario A. Gemelli IRCCS, Rome, Italy; ^5^AstraZeneca SpA, Milano Innovation District (MIND), Milano, Italy

**Keywords:** Healthentia, remote patient monitoring (RPM), digital therapeutics (DTx), software as medical device (SaMD), chronic diseases

## Abstract

**Background:**

Software as a Medical Device (SaMD) and mobile health (mHealth) applications have revolutionized the healthcare landscape in the areas of remote patient monitoring (RPM) and digital therapeutics (DTx). These technological advancements offer a range of benefits, from improved patient engagement and real-time monitoring, to evidence-based personalized treatment plans, risk prediction, and enhanced clinical outcomes.

**Objective:**

The systematic literature review aims to provide a comprehensive overview of the status of SaMD and mHealth apps, highlight the promising results, and discuss what is the potential of these technologies for improving health outcomes.

**Methods:**

The research methodology was structured in two phases. In the first phase, a search was conducted in the EuropePMC (EPMC) database up to April 2024 for systematic reviews on studies using the PICO model. The study population comprised individuals afflicted by chronic diseases; the intervention involved the utilization of mHealth solutions in comparison to any alternative intervention; the desired outcome focused on the efficient monitoring of patients. Systematic reviews fulfilling these criteria were incorporated within the framework of this study. The second phase of the investigation involved identifying and assessing clinical studies referenced in the systematic reviews, followed by the synthesis of their risk profiles and clinical benefits.

**Results:**

The results are rather positive, demonstrating how SaMDs can support the management of chronic diseases, satisfying patient safety and performance requirements. The principal findings, after the analysis of the extraction table referring to the 35 primary studies included, are: 24 studies (68.6%) analyzed clinical indications for type 2 diabetes mellitus (T2DM), six studies (17.1%) analyzed clinical indications for cardiovascular conditions, three studies (8.7%) analyzed clinical indications for cancer, one study (2.8%) analyzed clinical indications for chronic obstructive pulmonary disease (COPD), and one study (2.8%) analyzed clinical indications for hypertension. No severe adverse events related to the use of mHealth were reported in any of them. However, five studies (14.3%) reported mild adverse events (related to hypoglycemia, uncontrolled hypertension), and four studies (11.4%) reported technical issues with the devices (related to missing patient adherence requirements, Bluetooth unsuccessful pairing, and poor network connections). For what concerns variables of interest, out of the 35 studies, 14 reported positive results on the reduction of glycated hemoglobin (HbA1c) with the use of mHealth devices. Eight studies examined health-related quality of life (HRQoL); in three cases, there were no statistically significant differences, while the groups using mHealth devices in the other five studies experienced better HRQoL. Seven studies focused on physical activity and performance, all reflecting increased attention to physical activity levels. Six studies addressed depression and anxiety, with mostly self-reported benefits observed. Four studies each reported improvements in body fat and adherence to medications in the mHealth solutions arm. Three studies examined blood pressure (BP), reporting reduction in BP, and three studies addressed BMI, with one finding no statistically significant change and two instead BMI reduction. Two studies reported significant weight/waist reduction and reduced hospital readmissions. Finally, individual studies noted improvements in sleep quality/time, self-care/management, six-minute walk distance (6MWD), and exacerbation outcomes.

**Conclusion:**

The systematic literature review demonstrates the significant potential of software as a medical device (SaMD) and mobile health (mHealth) applications in revolutionizing chronic disease management through remote patient monitoring (RPM) and digital therapeutics (DTx). The evidence synthesized from multiple systematic reviews and clinical studies indicates that these technologies, exemplified by solutions like Healthentia, can effectively support patient monitoring and improve health outcomes while meeting crucial safety and performance requirements. The positive results observed across various chronic conditions underscore the transformative role of digital health interventions in modern healthcare delivery. However, further research is needed to address long-term efficacy, cost-effectiveness, and integration into existing healthcare systems. As the field rapidly evolves, continued evaluation and refinement of these technologies will be essential to fully realize their potential in enhancing patient care and health management strategies.

## 1 Introduction

### 1.1 Background

Software as a Medical Device (SaMD) and mobile health (mHealth) applications have revolutionized the healthcare landscape in the areas of remote patient monitoring (RPM) and digital therapeutics (DTx). These technological advancements offer a range of benefits, from improved patient engagement and real-time monitoring to personalized treatment plans and enhanced clinical outcomes.

The current landscape of SaMD and mHealth apps is characterized by rapid innovation and widespread adoption, especially after the Covid pandemic, which might have worked as a catalyst for digital health transformation. SaMD refers to software intended to be used for medical purposes without being part of a hardware medical device. mHealth applications; on the other hand, they encompass a wide range of digital tools designed to support health and wellness through mobile devices. mHealth applications are not necessarily medical devices and, therefore, they often do not comply with the regulatory framework. Both SaMD and mHealth apps have seen significant advancements, driven by the increasing need for accessible, efficient, and patient-centric healthcare solutions. Regulatory frameworks have also evolved to keep pace with these innovations, ensuring that these technologies meet the rigorous safety and efficacy standards. While the potential of SaMD and mHealth apps are immense, there are several challenges that need to be addressed to completely realize their benefits, such as data privacy concerns, integration with existing healthcare systems, and user adoption barriers.

The industrial outlook for remote patient monitoring (RPM) solutions shows significant growth potential as the increasing prevalence of chronic diseases such as diabetes, hypertension, and heart disease necessitate continuous monitoring. Advances in wearable technology and biosensors have revolutionized the RPM landscape, offering accurate, user-friendly, and cost-effective solutions. The shift toward value-based care models, which emphasize patient outcomes and cost efficiency over service volume, aligns with the capabilities of RPM systems. Investment trends indicate robust financial backing for RPM innovations and market forecasts predict sustained growth, with the global RPM market projected to expand significantly over the next decade. This expansion is supported by the rising adoption of telemedicine, advancements in artificial intelligence and data analytics, and the growing consumer demand for personalized healthcare solutions (1, 2).

### 1.2 Healthentia SaMD

Healthentia is a software intended for: a) the collection and transmission of physiological data including heart rate, blood pressure, oxygen saturation, and weight directly to care providers via automated electronic means in combination with validated IoT devices; b) the visualization (subjects-based dashboards) and the mathematical treatment of data (trends analysis, alerts) related to the monitored chronic disease subject’s physiological parameters; c) the transmission of patient’s outcomes and outcome scores related to patient’s health status, health-affecting factors, health-related quality of life, disease knowledge and adherence to treatment through validated questionnaires; d) the user (subject/patient) interaction with a conversational virtual coach for informative and motivational purposes, in order to support subject telemonitoring, decision making and virtual coaching (52, 53).

The Healthentia platform and its two front-end applications, the Healthentia mobile application for the subjects and the portal application for the healthcare professionals, is a standalone software [Software as a Service (SaaS)] medical active device. The platform consists of a collection of medical and non-medical modules. Medical modules are intended to collect, visualize, and compute patient’s physiological parameters to support the monitoring of the patient, decision-making during clinical trial, or under a medical treatment context. It is considered as a medical device because it is a software intended by the manufacturer to be used for diagnosis, prevention, monitoring, risk prediction, or prognosis of disease and does not achieve its principal intended action by pharmacological, immunological, or metabolic means.

The Healthentia platform is currently classified as Class IIa device per Rule 11 of Annex VIII of the Medical Device Regulation 2017/745 as amended. Based on the medical modules of Healthentia, healthcare professionals (HCPs) can monitor patients, causing an increased follow-up of the patients, which leads to good adherence to treatment.

Gathering information on lifestyle in a robust manner and collecting physiological parameters in real time continuously allows lifestyle elements to be aggregated into actionable inputs for diagnostic and patient management support.

**Figure 1:**
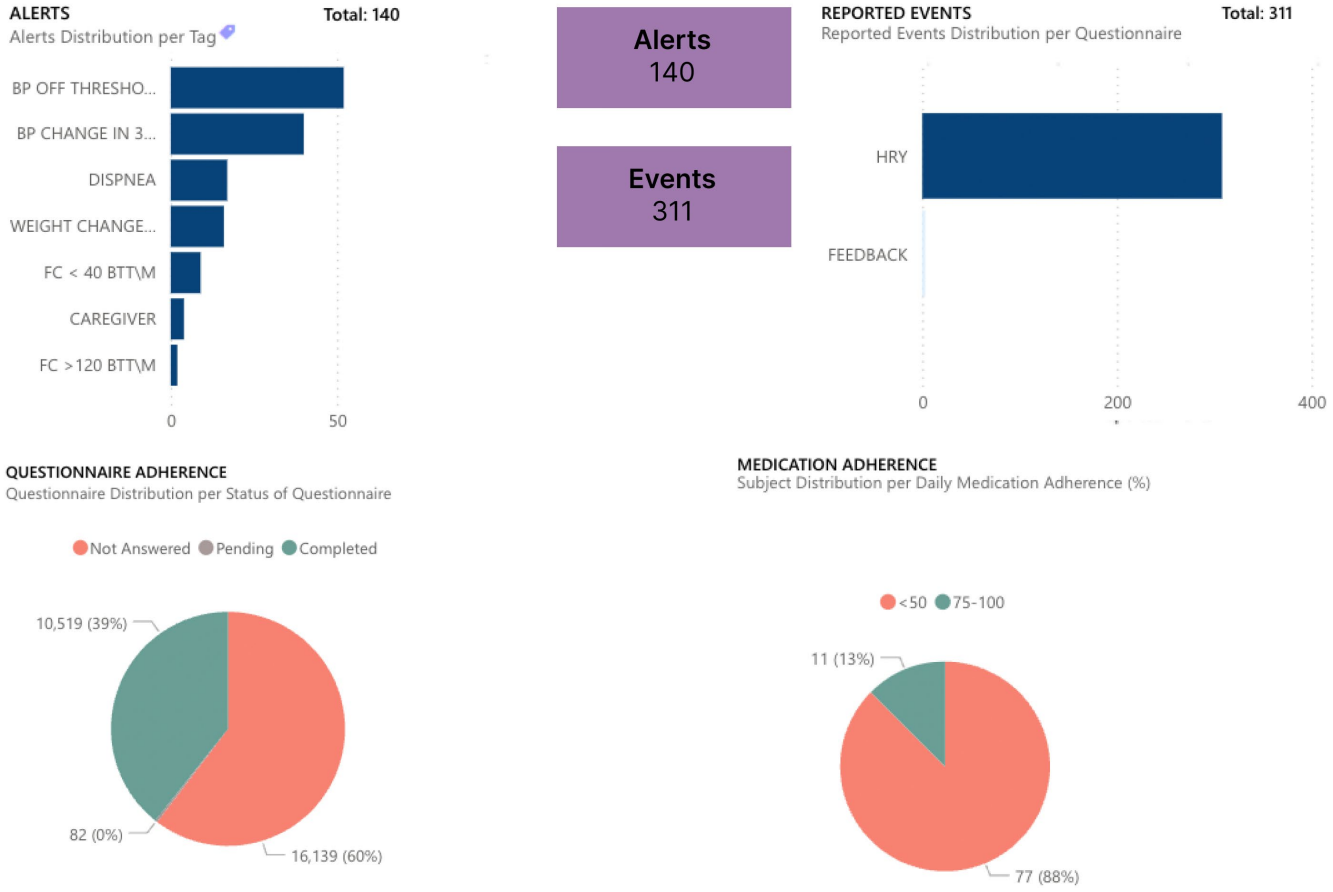
Dashboard for follow-up monitoring of patients.

**Figure 2:**
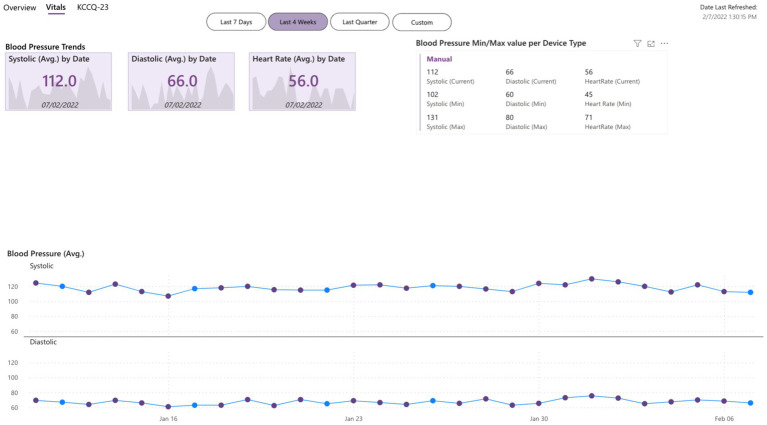
Dashboard of objective inputs for healthcare professionals.

The gathered data are more objective than feedback given by the patient at one moment (during an office or virtual appointment with HCPs) because they are quantifiable data (e.g., heart rate) over a long period. Moreover, these data are reported in graphs by Healthentia, which are objective and more easily interpretable by the HCPs, allowing a more evidence-based individualized decision-making, as presented in Figures 1, 2.

Healthentia is compatible with different supported devices to collect lifestyle information and vital signs, as grouped in Table 1 based on the objective data and their source.

**Table 1:** Objective data from patients.

Type	Measurements	Source
Activity	Steps walked, distance traveled, calories burned, floors climbed, minutes in different activity intensity zones	Activity trackers
	Exercise sessions (type, start time, duration, calories)	Activity trackers
Sleep	Sleep start, sleep end, minutes in different sleep zones	Activity trackers
Vitals	Blood pressure	Integrated Internet of Things (IoT) devices
	spO_2_	Integrated IoT devices
	Weight	Integrated IoT devices
	Heart (resting heart rate, max heart rate, minutes in different heart rate zones, heart rate variability)	Activity trackers

Healthentia indicates whether the Internet of Things (IoT) device has acceptable accuracy for the intended purpose or if it does not have acceptable accuracy and can only be used for measurements that do not require accuracy (e.g., step counter, sleep). The accuracy requirements for IoT devices that are connected to Healthentia are listed in Table 2.

**Table 2:** Minimum accuracy of devices.

Measurement	Min. accuracy
Blood pressure	≤10 mmHg (at least 85% probability)
spO_2_	Arms ±2–3% of arterial blood gas values
Heart (resting heart rate (RHR), max)	±10% of the input rate or ± 5 bpm (beats per minute)
Weight	±0.5–1.0 kg
Physical activity (steps)	n/a
Sleep	n/a

These devices constitute a safe combination and currently there is no device-specific information on any known restrictions to combinations. Furthermore, trend analysis is done by plotting the data collected over a period. This is made possible by Healthentia and supports a diagnostic decision for the healthcare professional.

**Figure 3:**
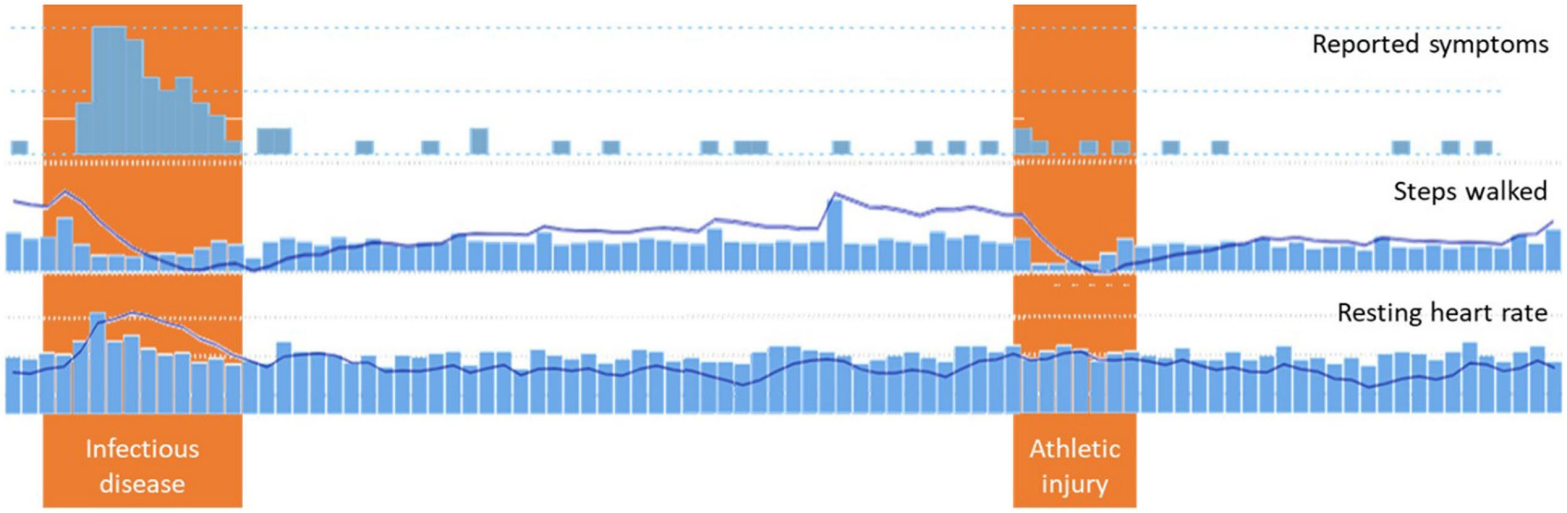
Trends analysis.

An example of trend analysis is shown in Figure 3, which contains screenshots of reported symptoms such as volume (indication of study commitment), measurements of steps walked, and vital measurements such as the resting heart rate, as well as their trends. The reported period includes two adverse events: an infectious disease and an athletic injury. During both events, the trend of the steps indicates a severe drop from the walking habit. But the trend of the resting heart rate indicates a worsening of that vital parameter over the established value only for the case of the infectious disease, as this vital parameter is not affected by the injury. In times other than the two events, the trends slowly recover to indicate the expected situation (normality) for the given patient.

Healthentia further to the RPM capabilities described above; it is an advanced DTx solution that aims to improve patients’ lifestyle using a novel behavioral change framework that includes techniques like goal setting and monitoring, offering visualizations of the patients’ data, and discussing aspects of risky behaviors with them. The behavioral change techniques use the chatbot functionality to provide a virtual coaching experience to the patients. While the dialogs are selected when certain conditions arise, they are personalized, since the content delivered is augmented with actual patient data. The data used for personalization can be simple, for example, static pieces of information like the patient’s name or sex. It can also be some personalized goals set for the patient by a doctor. Finally, it can be the results of statistics on some data of the patient, like the average steps walked in the past week, or the frequency red meat is consumed in the past month. This way actual patient data can be compared to the personalized goals, while the patient is addressed by name. Employing the existing features in the dialogs and looking ahead to more dynamic options, Healthentia can keep the patients interested, since the dialogs are dynamic, personalized, and offer two-way exchange of information, both from Healthentia to the patient, but also from the patient to Healthentia.

### 1.3 Objectives

During the continuous design, development, and validation of the medical device Healthentia, a systematic literature review has been conducted to assess the status and efficacy of these digital health interventions and benchmark the results with findings and clinical evidence collected from various studies.

The systematic literature review involved a scientific process of identifying, selecting, and analyzing relevant studies that evaluate the effectiveness of SaMD and mHealth apps in managing chronic diseases, using the PICO (Population, Intervention, Comparison, Outcomes and Study) framework. The selected studies provide a robust evidence base, highlighting the positive impact of these technologies on patient outcomes.

The systematic literature review provides a comprehensive overview of the current status of SaMD and mHealth apps, highlights promising results, and discusses the potential these technologies hold for improving health outcomes.

## 2 Methods

### 2.1 Search strategy

**Figure 4:**
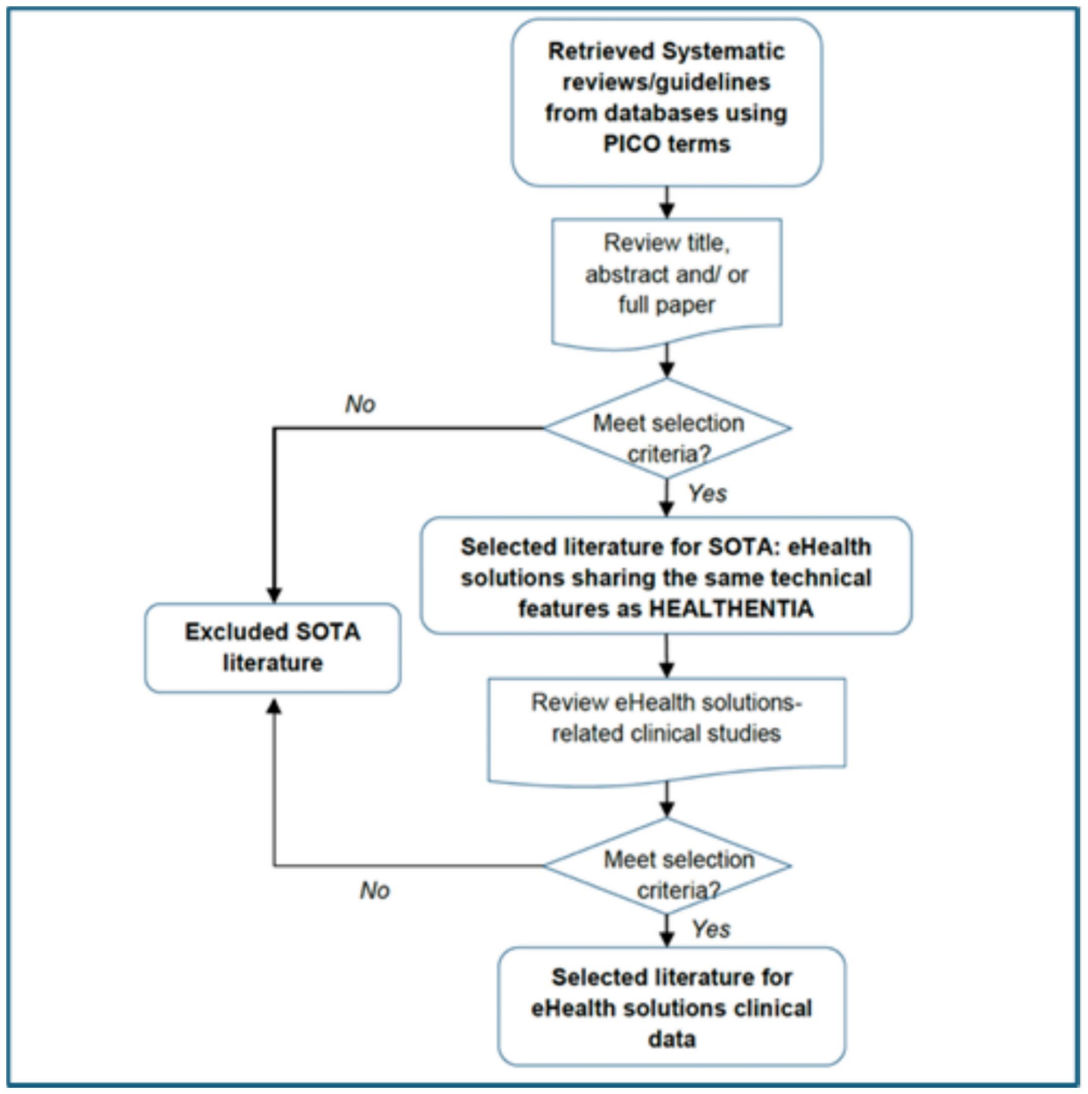
Strategy for state-of-the-art (SOTA) literature retrieval and screening process.

The research methodology was structured following a systematic approach as illustrated in Figure 4. Initially, a search was conducted in EuropePMC database up to April 2024 using the PICO model Schardt et al. (54):

- P—Patient, Population, or Problem: The study population comprised individuals afflicted with chronic diseases.
- I—Intervention or Exposure: The intervention involved the utilization of mHealth solutions.
- C—Comparison or Control: The comparison is to any alternative intervention.
- O—Outcome: The desired outcome focused on the efficient monitoring of patients.

The search protocol was not limited to EuropePMC and its full description was:

- Search on EuropePMC (52)
- Appraise the detected articles
  - If data are not sufficient, extend the search to Cochrane.
  - If data are still not sufficient, extend the search to PubMed with a focus on “mHealth” and publications for the last 3 years.
  - If data are still not sufficient, use alternative terms for the technology or the medical subject headings (MeSH) term “Telemedicine.”
  - If data are still not sufficient, search for clinical studies and not only for systematic reviews.

Regarding the subject-specific databases, there were no such databases considered, as the target was a broad scope of indications.

The systematic reviews fulfilling these criteria were incorporated within the framework of this study, which is presented in Figure 4. Five different queries were formulated concerning five specific chronic conditions, diabetes, chronic obstructive pulmonary disease (COPD), cancer(s), heart failure, and cardiovascular diseases:

- Search query for diabetes: *((“e-Health” OR “mHealth” OR “RPM” OR “Telehealth” OR “Internet of Things”) AND (TITLE:diabetes) AND (TITLE:"Systematic Review”))*
- Search query for COPD: *((“e-Health” OR “mHealth” OR “RPM” OR “Telehealth” OR “Internet of Things”) AND (TITLE:COPD) AND (TITLE:"Systematic Review”))*
- Search query for cancer: *((“e-Health” OR “mHealth” OR “RPM” OR “Telehealth” OR “Internet of Things”) AND (TITLE:cancer) AND (TITLE:"Systematic Review”))*
- Search query for heart failure: *((“e-Health” OR “mHealth” OR “RPM” OR “Telehealth” OR “Internet of Things”) AND (TITLE:"heart failure”) AND (TITLE:"Systematic Review”))*
- Search query for cardiovascular diseases: *((“e-Health” OR “mHealth” OR “RPM” OR “Telehealth” OR “Internet of Things”) AND (TITLE:"cardiovascular”) AND (TITLE:"Systematic Review”))*

The second phase of the investigation involved identifying and assessing clinical studies referenced in the Systematic Reviews, followed by the synthesis of their risk profiles and clinical benefits.

### 2.2 Inclusion and exclusion criteria

The inclusion criteria for the studies involved in this research focused on monitoring chronic diseases using devices similar to Healthentia, with a control group for comparison to gather clinical data. Studies primarily centered on economic aspects without relevance to mHealth solutions or chronic diseases were excluded from consideration, as indicated in Table 3.

**Table 3:** Inclusion and exclusion criteria.

PICO reference	Inclusion criteria	Exclusion criteria
Population	Chronic disease monitoring	Other pathologies management
Intervention	Report use of similar devices	Evaluation of other management methods without comparison to e-Health solution
Comparison	Comparative study (with control group)	NA
Outcome	Reported clinical benefits for patients	Economic considerations

### 2.3 Screening of articles

Concerning the screening of the articles, the title, abstract and full article, as applicable, were screened and verified for meeting the inclusion and exclusion selection criteria. In case the selection criteria are met, the articles are included to establish the state-of-the-art. When the included articles contained clinical data on e-health solutions substantiated by referenced articles, those publications were screened to retrieve detailed clinical study information (clinical indications, number of patients, device used, performance data, and side-effects). Screening was performed by title, abstract, and full article analysis based on the selection criteria. In case the selection criteria were met, the clinical studies containing performance and safety data were included for analysis.

### 2.4 Study selection

Four independent researchers (S.K., A.P., L.F., K.K.) initially screened the titles and abstracts resulting from the five aforementioned queries. Subsequently, pertinent full texts from the previously accepted studies were incorporated by the same team. Following this inclusion, a hand search for device-related studies within the references of the systematic reviews identified during the initial screening was conducted. The key findings were mainly randomized control trials assessing mHealth solutions with functionalities similar to those of Healthentia.

### 2.5 Data extraction

Two extraction tables were developed as part of the research process. The first extraction table, concerning systematic reviews resulting from the search queries, contained essential details such as title, authors, journal of publication, publication year, DOI, along with clinical performance data and conclusions collected from meta-analyses. Following the manual search process mentioned earlier, a second extraction table was created to compile more specific information from the referenced primary studies. This table included details such as title, authors, publication year, DOI, clinical indication, study type, device type and name, patient numbers in both device and control groups, as well as data on HbA1c (glycated hemoglobin), blood pressure, LDL-c (low-density lipoprotein cholesterol), (HR) QoL (health-related quality of life), weight/waist measurements, exacerbations, 6MWD (six-minute walk distance), adherence rates, hospital readmissions, self-care/management practices, depression and anxiety assessments, physical activity levels, sleep patterns, adverse events, complications, and conclusions drawn from the gathered information.

### 2.6 Limitations of the study

The methodological decision to exclusively gather systematic reviews derived from the search queries conducted on a single database was a necessary choice driven by time limitations. Nonetheless, this approach may have introduced a potential selection bias, given that the scope of studies available for data collection was inevitably narrower in comparison to the broader spectrum present in the literature, considering the major interest in the findings of primary studies that collected the necessary information.

## 3 Results

### 3.1 Characteristics of the included studies

**Figure 5:**
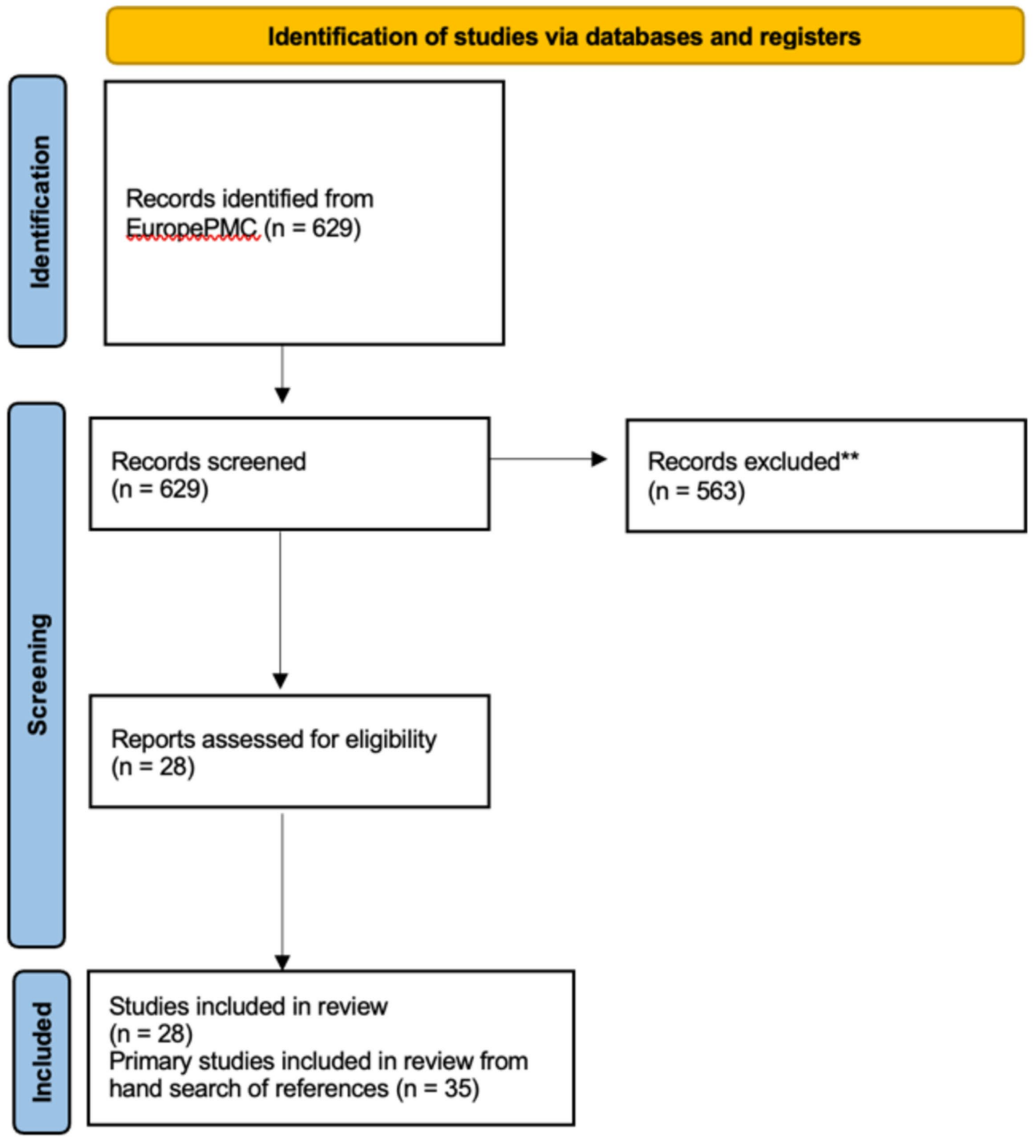
Flow chart of literature search [Preferred Reporting Items for Systematic Reviews and Meta-Analyses (PRISMA) 2020].

The EuropePMC research resulted in 629 records. Following the screening of titles and abstracts, 563 records were deemed irrelevant and excluded. Subsequently, 28 systematic reviews were included after a full-text examination. Within these 28 records, references were reviewed manually to identify key primary studies on mHealth solutions. Initially, 116 sets of clinical data were considered relevant. However, after a detailed review of full texts, the number of pertinent studies was refined down to 35 (see Figure 5).

Table 4 summarizes data extracted from the first step of the search strategy (systematic reviews, results from meta-analysis: 14 records) and Table 5 summarizes data extracted from the second step of the search strategy (primary studies: 35 records).

**Table 4:** Clinical performance of eHealth solutions (systematic reviews/meta-analyses results).

References	Condition	Clinical performance	Conclusions
de Souza et al. (3)	Type II diabetes (T2DM)	HbA1c: Use of mobile applications reduced HbA1c in 0.39% (CI 0.24–0.54)	The use of mobile applications can help reduce glycated hemoglobin (HbA1c) by 0.39% compared to the usual care group
Lee et al. (4)	Type II diabetes	Significant benefits: Glycosylated hemoglobin (HbA1c) (mean difference −0.24%; 95% confidence interval [CI]: −0.44, −0.05; *p* = 0.01) Postprandial blood glucose (−2.91 mmol/L; 95% CI: −4.78, −1.03; *p* = 0.002) Triglycerides (−0.09 mmol/L; 95% CI: −0.17, −0.02; *p* = 0.01) Non-significant impact on low-density lipoprotein cholesterol, high-density lipoprotein cholesterol, blood pressure (systolic blood pressure; diastolic blood pressure)	Among older adults with T2DM, mHealth interventions were associated with improved cardiometabolic outcomes versus usual care
Kim et al. (5)	Type II diabetes	Significant benefits: Decreased HbA1c (*p* < 0.00001, mean difference = −0.49) Non-significant impact on triglyceride decrease, BMI, total cholesterol, HDL-C, LDL-C, systolic BP or diastolic BP	Digital healthcare technology can improve HbA1c and triglyceride levels of type 2 diabetes patients
He et al. (6)	Type II diabetes	Significant benefits: Decreased HbA1c (mean difference, −0.45%; 95% CI, −0.58 to −0.32; *p* < 0.001) Improved medication adherence (0.80; 95% CI, 0.15–1.46; *p* = 0.02) Non-significant impact on psychological status, quality of life and cardiometabolic risk factors	Smartphone application–based diabetes self-management intervention could optimize patients’ glycaemic control and enhance participants’ self-management performance
Liu et al. (7)	Type II diabetes	Significant benefits: Reduction in HbA1c levels (mean difference −0.44, 95% CI −0.59 to −0.29; *p* < 0.001) Systolic blood pressure (SBP) (−0.17, 95% CI −0.31 to −0.03, *p* = 0.02) Diastolic blood pressure (DBP) (−0.17, 95% CI −0.30 to −0.03, *p* = 0.02)	Mobile app-assisted self-care interventions can be an effective tool for managing blood glucose and blood pressure, likely because their use facilitates remote management of health issues and data, provision of personalized self-care recommendations, patient–care provider communication, and decision-making
Masotta et al. (8)	Heart disease	Significant benefits in studies describing the remote transmission of patient’s physiologic parameters: Reduced number of patients rehospitalized at 1 year (RR = 0.40, 95% CI = 0.19–0.81; *p* < 0.05) Non-significant impact on probability of one-year all-cause mortality	The review confirms the benefits of telemonitoring in reducing rehospitalizations of heart failure (HF) patients
Kitsiou et al. (9)	Heart disease	Significant benefits: Reduced the risk of all-cause mortality (risk ratio [RR], 0.80; 95% confidence interval [CI], 0.65–0.97; absolute risk reduction [ARR], 2.1%; high-quality evidence) Reduced cardiovascular mortality (RR, 0.70; 95% CI, 0.53–0.91; ARR, 2.9%; high quality evidence) Reduced HF hospitalizations (RR, 0.77; 95% CI, 0.67–0.88; ARR, 5%; high-quality evidence) No effect on all-cause hospitalizations	mHealth interventions with remote monitoring and clinical feedback reduce mortality and HF-related hospitalizations, but might not reduce all-cause hospitalizations in patients with HF
Uminski et al. (10)	Heart disease	Significant benefits [post-discharge Virtual Ward (VW)] Reduced risk of mortality (RR 0.59, 95% CI = 0.44 ± 0.78) Reduced heart failure related readmissions (RR 0.61, 95% CI = 0.49 ± 0.76) All-cause readmission was not reduced Do not reduce death or hospital readmissions for patients with undifferentiated high-risk chronic diseases	A post-discharge VW can provide added benefits to usual community-based care to reduce all-cause mortality and heart failure-related hospital admissions among patients with heart failure
Jaén-Extremera et al. (11)	Heart disease	Significant benefits: Decreased HbA1c [mean difference −0.432 (95% CI: −0.522 to −0.341; *p* < 0.001)] Decreased systolic blood pressure [−0.775 (95% CI: −0.887 to −0.663; *p* < 0.001)] Decreased diastolic blood pressure [−0.447 (95% CI: −0.572 to −0.321; *p* < 0.001)] Overweight [−0.628 (95% CI: −0.739 to −0.517; *p* < 0.001)]	For all the risk factors, a small effect of the telemedicine was seen
Cruz-Cobo et al. (12)	Heart disease	Significant benefits: Exercise capacity measured using the 6-min walk test (mean difference = 21.64, 95% CI 12.72–30.55; *p* < 0.001) Physical activity (0.42, 95% CI 0.04–0.81; *p* = 0.03) Adherence to treatment (risk difference = 0.19, 95% CI 0.11–0.28; *p* < 0.001) Physical and mental dimensions of quality of life were better in the mHealth group (0.26, 95% CI 0.09–0.44; *p* = 0.004 and 0.27, 95% CI 0.06–0.47; *p* = 0.01, respectively) Hospital readmissions for all causes and cardiovascular causes were statistically higher in the control group than in the mHealth group (−0.03, 95% CI –0.05 to −0.00; *p* = 0.04 vs. −0.04, 95% CI –0.07 to −0.00; *p* = 0.05)	mHealth technology has a positive effect on patients who have experienced a coronary event in terms of their exercise capacity, physical activity, adherence to medication, and physical and mental quality of life, as well as readmissions for all causes and cardiovascular causes
Patterson et al. (13)	Heart disease	Significant benefits: Increase of 40.35 min of moderate-to-vigorous intensity physical activity per week (*p* = 0.04; 95% CI 1.03 to 79.67)	Smartphone applications were effective in increasing physical activity in people with cardiovascular disease. Caution is warranted for the low-quality evidence, small sample, and larger coronary heart disease representation
Lu et al. (14)	COPD	Significant benefits: Reduce emergency room visits [mean difference −0.70, 95% CI −1.36 to −0.03] Reduced exacerbation-related readmissions (risk ratio 0.74, 95% CI 0.60–0.92) Reduced exacerbation-related hospital days (MD −0.60, 95% CI −1.06 to −0.13) Reduced mortality (odds ratio 0.71, 95% CI 0.54–0.93) No difference with respect to all-cause readmissions, the rate of exacerbation-related readmissions, all-cause hospital days, time to first hospital readmission, anxiety and depression, and exercise capacity	The implementation of telemedicine intervention is a potential protective therapeutic strategy that could facilitate the long-term management of acute exacerbation of chronic obstructive pulmonary disease (AECOPD)
Jang et al. (15)	COPD	Significant benefits: Decreased the number of emergency room (ER) visits due to severe exacerbations [mean difference = −0.14; 95% CI: −0.28, −0.01] Do not reduce the number of admissions. No benefit in mortality, quality of life, or cost-effectiveness.	Adding telemonitoring to the usual care for chronic obstructive pulmonary disease (COPD) may reduce unnecessary ER visits but is unlikely to prevent hospitalizations from COPD exacerbations
Buneviciene et al. (16)	Cancer	Significant benefits: Improvement of EORTC QLQ-C30 Global Health Status (mean difference: 8.48; 95%CI: 4.16; 12.8; *p* < 0.01) Improvement of Short Form Survey Instrument (SF-36) (15.4; 95 %CI: 5.30; 25.5; *p* < 0.01) scores (1 study) No improvement of the Functional Assessment of Cancer Therapy—General (FACT-G) scores (−0.03; 95%CI: −0.19; 0.13; *p* < 0.01)	The majority of studies (15 randomized controlled trials (RCTs) and 7 pre-post designs) found that mHealth interventions were associated with improvement in at least one domain of health-related quality of life (HRQoL) of cancer patients

**Table 5:** Clinical performance of eHealth solutions (references of systematic reviews included).

Reference	Clinical indication	Study type	Device under evaluation	Patients (eHealth solution)	eHealth solution—clinical performance	Side-effects, complications related to eHealth solution	Conclusions
Holmen et al. (17)	Type 2 diabetes (T2DM)	Prospective randomized controlled trial (RCT)	Few Touch Application (FTA) (diary app, glucometer, food habit registration system, physical activity registration system, a personal goal setting system, and a general information system)	39 to the FTA group, 40 to the FTAhealth counseling (FTA-HC) group	HbA1c level decreased in all groups, but did not differ between groups after 1 year. The mean change in the heiQ domain skills and technique acquisition was significantly greater in the FTA-HC group after adjusting for age, gender, and education (*p* = 0.04)	No serious adverse clinical events reported during the study period Technical issues like Bluetooth pairing problems were reported but not severe	HbA1c level decreased in all groups but did not differ FTA-HC group showed significantly greater improvement in skills and techniques No significant differences in secondary outcomes between groups after 1 year
Karhula et al. (18)	T2DM	RCT	eClinic (Medixine Ltd) [information content, health parameters registered by the corresponding measurement devices, personal care plan entered by the health coach in agreement with the patient, and data obtained from the electronic health record (EHR)]	175 heart patients and 162 diabetes patients	(HR)QoL: no statistically significant benefits over the current practice with regard to health-related quality of life—heart disease patients Weight/waist: significant difference in waist circumference in the type 2 diabetes group (beta = −1.711, *p* = 0.01)	None	Health coaching with telemonitoring did not improve quality of life or clinical condition Positive changes in clinical conditions were observed in both study groups Lack of consistency in variables suggests the significant difference may be false
Li et al. (19)	T2DM	RCT	R Plus Health app and chest strap for physical activity	44	HbA1c level: No significant difference BMI: −0.60 kg/m^2^ (device) vs. −0.32 (control), but not statistically significant Physical activity and performance: −2.0 bpm (device) vs. 1.0 bpm (control)	No severe adverse events found in either group Hypoglycemia occurred 4 times in the intervention group and 8 times in the control group Complications included uncontrolled hypertension, hyperthyroidism, and osteoarthritis	Intervention group showed better cardiopulmonary endurance and body fat reduction No significant difference in hemoglobin A1c level reduction between groups More participants in intervention group reduced or stopped antidiabetic drugs Wearable technology ensures accurate assessment of exercise duration and intensity
Coombes et al. (20)	T2DM	RCT	Personal activity Intelligence Health app and wrist-worn heart rate monitor	12	Body fat: Significant improvements in body fat: −1.3% Physical activity and performance: Significant improvements in exercise capacity: +63 PAI Sleep: Significant improvements in sleep time: +67.2 min	None A mild hypoglycemic episode	PAI e-Health Program is feasible, acceptable, and efficacious in T2D Participants achieved Personal Activity Intelligence (PAI) score ≥ 100 on 56.4% of days (mean 119.7) Significant improvements in exercise capacity, sleep time, and body composition
Boer et al. (21)	COPD	RCT	Living Well with COPD [pulse oximeter, spirometer, forehead thermometer, questionnaires, advice (coaching)]	36	Exacerbations: no statistically significant differences between the intervention group and the control group in exacerbation-free weeks (mean 30.6, SD 13.3 vs. mean 28.0, SD 14.8 weeks, respectively; rate ratio 1.21; 95% CI 0.77–1.91)	None	No beneficial effects of mHealth tool on COPD exacerbation-free time Patients valued mHealth tool’s supportive function and usability mHealth may be a valuable alternative for COPD patients
Sun et al. (22)	T2DM	RCT	Glucometer connected via Bluetooth to mHealth app showing advice and reminders. Daily diet info.	44	HbA1c level: 6 months: 6.84% (device) vs. 7.22% (control) Self-care/ management: Self reported improved the self-monitoring of patients’ blood glucose levels (0.93), diet, exercise, and other self-management skills (0.85), and knowledge of diabetes (0.98) Depression & anxiety: Self-reported improved effect on their psychological status (0.96)	None	Intervention group had significantly lower Porphobilinogen levels after 3 months Improved glycaemic control was sustained and significantly different from the control group Enhanced communication, real-time tracking, and patient compliance contributed to improved outcomes Telemedicine was deemed effective and safe for older diabetic patients
Chao Dyna et al. (23)	T2DM	RCT	Custom/unspecified app with questionnaires	49	HbA1c level: −1.52 (device) vs. −1.13 (control) Weight: −10.4 (device) vs. −1.02 (control) Self-care/management: Improved health knowledge 4.92 (device) vs. −1.56 (control). Improved behavior compliance. Compliance rate higher in women, steady, and dominant individuals. Better eating and monitoring Physical activity and performance: Increased attention to activity	None	Mobile app interventions improved patient compliance and health knowledge Age did not hinder mobile device usage for wellness education Personalized interactive education led to greater compliance with health behaviors Compliance rate increased after IPMF-based educational intervention
Huang et al. (24)	T2DM	Randomized two-arm pre-posttest control group	Medisafe app with questionnaires and reminders	22	HbA1c level: No improvement BMI: Higher decrease in device (−4.2) than control (−0.5) adherence: Lower self-reported barriers to medication adherence: decrease for device (−1.4) vs. increase for control (3) Self-care/management: Improved awareness	None	Feasibility of smartphone app for medication adherence in Asian diabetes patients Improved awareness, reduced barriers to medication adherence in intervention group No significant improvement in HbA1c level observed in the study
Zhai and Yu (25)	T2DM	RCT	Yutangyihu app: Connected glucose meter and diet advice, emotional management, and medication guidance	60	HbA1c level: Higher reduction in device (−1.95) than control (−1.83) Self-care/ management: Much greater increase of self-efficacy score in device (30.7) than control (12.9)	None	Mobile app improved HbA1c control and self-efficacy in patients App enhanced therapeutic outcomes and self-management in type 2 diabetes App experiences can aid in preventing and managing other chronic diseases
Bailey et al. (26)	TD2M	Randomized, controlled feasibility study	MyHealthAvatar-Diabetes app	9	Body fat: Improvements in body fat % Physical activity and performance: Improvements in sitting breaks	Technical issues affected app adherence and interest among participants	Feasible to deliver and evaluate MyHealthAvatar- Diabetes app for health outcomes Preliminary improvements in sitting breaks, body fat %, and glucose tolerance App viewed as acceptable for reducing sitting time and improving health
Patnaik et al. (27)	T2DM	RCT	Custom/ unspecified app	33	Weight/BMI/waist: Significant decrease in weight, BMI, waist circumference, hip circumference, body fat percentage, and SBP in the intervention group. The mean BMI of the study participants was 27.47 ± 4.34 kg/m^2^. Both control and intervention groups contain two (6.1%) normal patients while overweight patients were more in the control group (78.8%) than intervention group (69.7%) Physical activity and performance: Higher physical activity levels in intervention group met WHO recommendations	None	Cost-effective mobile apps encourage physical activity in diabetes patients Mobile applications intervention program on healthy lifestyle significantly improves the weight, BMI, waist circumference, hip circumference, body fat percentage, blood pressure, and physical activity among the type II diabetes patients
Park et al. (28)	Advanced lung cancer undergoing chemotherapy	Pilot study	Aftercare app (questionnaires, chat, notifications, wearable device, portable pulse oximeter, thermometer, scale, and resistance bands for physical therapy)	100	6MWD: Significant improvement in the 6MWD; 380.1 m (SD 74.1) at baseline, 429.1 m (SD 58.6) at 6 weeks (*p* < 0.001), and 448.1 m (SD 50.0) at 12 weeks (*p* < 0.001). Depression and anxiety: Role (*p* = 0.02), emotional (*p* < 0.001), and social functioning (*p* = 0.002) scale scores showed significant improvement after PR	Not significant	Improved exercise capacity, symptom management, and quality of life Dyspnoea scale showed no significant improvement overall Significant improvement in role, emotional, and social functioning scale scores Symptom scale scores for fatigue, anorexia, and diarrhea improved significantly No significant change in quality of life and severity of pain
Lim et al. (29)	T2DM	RCT	u-healthcare website	50	HbA1c level: HbA1c levels decreased significantly in the u-healthcare group [8.0 ± 0.7% (64.2 ± 8.8 mmol/mol) to 7.3 ± 0.9% (56.7 ± 9.9 mmol/mol)] compared with the SMBG group [8.1 ± 0.8% (64.9 ± 9.1 mmol/mol) to 7.9 ± 1.2% (63.2 ± 12.3 mmol/ mol)] (*p* < 0.01). Proportion of patients with HbA1c < 7% was higher in u-healthcare group Body fat mass decreased, and lipid profiles improved in u-healthcare group 18.4 (6.9)—SMBG 20.4 (7.2)	Not significant	U-healthcare service effectively managed older patients with type 2 diabetes Instant feedback and recommendations were more effective than self-measuring glucose levels
Pappot et al. (30)	Cancer	Interventional Study	Kræftværket app (questionnaires)	10 (active treat.) 10 (post-treat.)	(HR)QoL: Significant increase in overall QoL after the 6-week period (global QoL: baseline 62.5, SD 22.3; after 6 weeks 80.8, SD 9.7; *p* = 0.04)	None	This study shows the feasibility and possible effect on QoL associated with the use of an mHealth tool in AYA patients
Yang et al. (31)	Cancer	RCT	Pain Guard app (questionnaires, reminders, reports, e-diary, real-time medication consultation, content)	27	(HR)QoL: No significant differences in baseline pain scores or baseline QoL scores between groups. Improvements in global QoL scores in the trial group were also significantly higher than those in the control group (*p* < 0.001)	Adverse reactions in the trial group (7/31) were lower than that in the control group (12/27), especially constipation, with significant differences (*p* = 0.01)	Pain Guard was effective for the management of pain in discharged patients with cancer pain, and its operability was effective and easily accepted by patients
Hansel et al. (32)	T2DM	RCT	Web-based program called Accompagnement Nutritionnel de l’Obésité et du Diabète (ANODE)	52	Self-care/management: Changes in dietary intake tended to differ between arms for lipids (*p* = 0.02), saturated fats (*p* < 0.01), sodium (*p* = 0.07), and empty calories (*p* = 0.06), always toward healthier foods in the intervention arm Depression and anxiety: Completers differed from non-completers in antihypertensive drug prescription frequency	Participants’ connections varied, with decreasing logins throughout the study	ANODE e-coaching program improved diet quality and cardiometabolic risk factors significantly
Katalenich et al. (33)	T2DM	RCT	Diabetes Remote Monitoring and Management System (DRMS)	50	(HR)QoL: Difference between the DQoL– Social/Vocational Concerns subscale scores was statistically significant 9.63 (DRMS) vs. 11.10 (control group); P ¼ 0.04 Self-care/ management: Improved diabetes management	Technical issues reported with system recognizing responses accurately System limitations with complex insulin adjustment algorithms for multiple injections	DRMS system showed similar glycaemic control to usual clinic care Further research needed to sustain benefits over longer periods
Nagrebetsk et al. (34)	T2DM	Feasibility trial in primary care	t Diabetes app	7	HbA1c level: Median change in HbA1c at 6 months was −10 mmol/mol in intervention group Self-care/management: Patients understood how to adjust medication based on blood glucose graphs	Tight glycaemic control reduces complications in type 2 diabetes patients Intensive glucose control impacts vascular outcomes and mortality in diabetes	Self-titration of oral glucose lowering medication using telehealth platform was feasible Patients showed improved glycaemic control with the stepwise treatment plan
Davoudi et al. (35)	HF	RCT	My Smart Heart (reminders, content, messages, frequently asked questions, daily recording of physical and psychological symptoms, vital signs, alerts)	55	(HR)QoL: Statistically significant differences in the mean scores of quality of life and its dimensions after the intervention, thereby indicating a better quality of life in the intervention group (*p* < 0.001)	None	Use of a smartphone-based app can improve the quality of life in patients with heart failure. The results of our study recommend that digital apps be used for improving the management of patients with heart failure
Clays et al. (36)	CHF	RCT	HeartMan (questionnaires, blood pressure monitor, weight scale, pill organizer, heart rate, galvanic skin response, skin temperature and activity tracking)	34	(HR)QoL: No significant intervention effects were observed for HRQoL, self-care confidence, illness perception and exercise capacity Self-care/management: Although the group differences were not significant, selfcare increased (*p* < 0.05), and sexual problems decreased (*p* < 0.05) in the intervention group only Depression and anxiety: All depression and anxiety dimensions decreased in the intervention group (*p* < 0.001), while the need for sexual counseling decreased in the control group (*p* < 0.05)	None	HeartMan system improved mental and sexual health in congestive heart failure (CHF) patients No significant effects on HRQoL, exercise capacity, and illness perception Future studies needed to validate HeartMan system effectiveness in larger samples
Athilingam et al. (37)	HF	Feasibility study–RCT	HeartMapp (questionnaires, daily weighing, symptom assessment, responding to tailored alerts, vital sign monitoring, content)	7	(HR)QoL: Quality of life declined among both groups, more so in the control group (2.14 vs. 9.0; *t*−1.43 = 11, *p* = 0.18) Self-care/ management: Participants in the HeartMapp group had a significant mean score change on self-care management (8.7 vs. 2.3; t3.38 = 11, *p* = 0.01), self-care confidence (6.7 vs. 1.8; t2.53 = 11, *p* = 0.28), and HF knowledge (3 vs. −0.66; t2.37 = 11, *p* = 0.04). Depression improved among both groups, more so in the control group (−1.14 vs. −5.17; t1.97 = 11, *p* = 0.07)	None	HeartMapp showed significant improvements in self-care management and HF knowledge
Park et al. (38)	HF	Feasibility study	RxUniverse prescribed HealthPROMISE and iHealth apps (content, reminders, questionnaires, blood pressure, weight)	60	Hospital readmissions: The 30-day hospital readmission rate was 10% (6/58), compared with the national readmission rates of approximately 25% and the Mount Sinai Hospital’s average of approximately 23%	None	Identified factors and trends for effective remote monitoring post-hospital discharge Real-time vital sign data interventions reduce readmissions and improve outcomes
Wang et al. (39)	T2DM	RCT	Custom/ unspecified app	60	HbA1c level: After the intervention, levels of FPG, 2-h postprandial blood glucose, and HbA1c were lower in the test group than in the control group; the differences were statistically significant (*p* < 0.05) Hospital readmissions: Reduced rehospitalization rates and average number of rehospitalizations post-discharge (−1.19 vs. *p* < 0.05) Self-care/management: Before the intervention, there were no statistically significant differences in disease awareness and self-management between the two groups (*p* > 0.05). After the 6-month intervention, both abilities were found to be higher in the test group than in the control group. This difference was statistically significant (*p* < 0.05)	None	Continuous care via mobile health app is beneficial for type 2 diabetes Mobile healthcare shows promise for improving patient knowledge and outcomes
Gunawardena et al. (40)	T2DM	RCT	Smart Glucose Manager (SGM)	27	HbA1c level: At the 6-month follow up, the SGM group had significant lower A1c levels than the control group (7.2% vs. 8.17%, *p* < 0.0001). For both groups, A1c values decreased from baseline to the 3 months (SGM: 9.52 to 8.16%, *p* < 0.0001; control: 9.44 to 8.31%, *p* < 0.0001). From 3 months to 6 months, the SGM group showed further improvement of A1c (−0.96% *p* < 0.0001), whereas the control group did not (*p* = 0.19). A1c improvement was positively correlated with SGM usage (*R* = 0.81, *p* < 0.001)	None	SGM app positively impacted diabetes management and A1 levels Further research needed to assess long-term compliance and app components
Kleinman et al. (41)	T2DM	RCT	Gather Health app	44	Adherence: Improved medication adherence (39.0% vs. 12.8%; *p* = 0.03) Self-care/ management: Increased frequency of blood glucose (BG) self-testing (39.0% vs. 10.3%; *p* = 0.01) at 6 months from baseline	None	m-Health led to increased medication adherence and blood glucose testing The tool can expand access to quality chronic disease care
Fukuoka et al. (42)	T2DM	RCT	mDPP mobile app	30	BP: Intervention group had reductions in blood pressure (*p* < 0.05) Weight: Intervention group lost 6.2 kg and had decreased hip circumference, control group gained 0.3 kg Self-care/management: Intervention group: reductions in intake of saturated fat (*p* < 0.007) and sugar-sweetened beverages (*p* < 0.02) Physical activity and performance: Intervention group increased steps by 2,551, control group decreased by 734	None	Mobile app intervention led to significant weight loss and lifestyle improvements The study suggests further investigation in a larger trial
Wayne et al. (43)	T2DM	RCT	Custom/unspecified app	48	HbA1c level: Both groups reduced HbA1c levels, with no significant between-group differences, but intervention group showed accelerated reduction at 3 months (HR)QoL: Both groups reported improvements in mood, satisfaction with life, and quality of life Weight: Intervention group participants also had significant decreases in weight (*p* = 0.006) and waist circumference (*p* = 0.01) while controls did not	None	Health coaching improved glucoregulation and mental health in lower-SES T2DM patients Mobile phone support accelerated HbA1c reduction in the intervention group Both groups showed improvements in mood, satisfaction with life, and quality of life Psychological wellbeing improved from baseline to 6-month follow-up
Pernille et al. (44)	Cardiac rehabilitation	RCT	Vett® (goal setting, reminders, questionnaires, weight, BP)	55	Physical activity and performance: Statistically significant difference in VO_2_ peak between the groups at follow-up of 2.2 mL/kg/min, 95% confidence interval 0.9–3.5 (*p* < 0.001). Statistically significant differences were also observed in exercise performance, exercise habits and in self-perceived goal achievement	None	Individualized follow-up for 1 year with an app significantly improved VO2peak, exercise performance and exercise habits, as well as self-perceived goal achievement, compared with a CG in patients post-CR. There were no statistically significant differences between the groups at follow-up in the other outcome measures evaluated
Johnston et al. (45)	MI	RCT	SUPPORT study (AstraZeneca) (e-diary, medication management, reminders, content, questionnaires)	85	Adherence: Greater patient registered drug adherence was achieved in the active vs. the control group (non-adherence score: 16.6 vs. 22.8 [*p* = 0.025]). Numerically, the active group was associated with higher degree of smoking cessation, increased physical activity, and change in quality of life; however, this did not reach statistical significance	None	Interactive patient support tool improved drug adherence and patient satisfaction Trend toward better cardiovascular lifestyle changes and quality of life observed Smartphone app is a promising aid for secondary prevention in MI patients
Persell et al. (46)	Hypertension	RCT	Hypertension Personal Control Program coaching app (questionnaires, blood pressure)	144	BP: Baseline mean (SD) systolic blood pressure was 140.6 (12.2) mmHg among intervention participants and 141.8 (13.4) mmHg among control participants. After 6 months, the corresponding mean (SD) systolic blood pressures were 132.3 (15.0) mmHg and 135.0 (13.9) mmHg, with a between-group adjusted difference of −2.0 mmHg (95%CI, −4.9 mm Hg to 0.8 mm Hg; *p* = 0.16). At 6 months, self confidence in controlling blood pressure was greater in the intervention group (0.36 point on a 5-point scale; 95%CI, 0.18 point to 0.54 point; *p* < 0.001)	None	Among individuals with uncontrolled hypertension, those randomized to a smartphone coaching app plus home monitor had similar systolic blood pressure compared with those who received a blood pressure tracking app plus home monitor. Given the direction of the difference in systolic blood pressure between groups and the possibility for differences in treatment effects across subgroups, future studies are warranted
Hilmarsdóttir et al. (47)	T2DM	RCT	SidekickHealth app	15	HbA1c level: The reduction of HbA1c in the intervention group was from 61 ± 21.4 mmol/mol at baseline to 52.7 ± 15.2 mmol/mol after 6 months. This indicates a decrease in HbA1c levels by approximately 8.3% Depression and anxiety: Significant decrease in disease-specific distress from 19.5 ± 16.5 to 11.7 ± 13.4, and in anxiety symptoms from 5.4 ± 4.0 to 4.1 ± 3.8	None	No significant difference in HbA1c reduction between the intervention and control groups SidekickHealth program Enhances T2DM outpatient treatment for glycaemic control Psychological wellbeing improvement noted in patients using the digital lifestyle program Larger confirmative studies are required to validate the program’s effectiveness
Lee et al. (48)	T2DM	RCT	Switch application	54	Self-care/management: Better self-management, maintained after intervention	None	Addition of Tailored Mobile Coaching to conventional diabetes management was shown to be effective in reducing HbA1c levels and improving diabetes self-management TMC system was effective, reproducible, and durable in diabetes management
Yu et al. (49)	T2DM	RCT	Diabetes-Carer	45	HbA1c level: At 24 weeks, the HbA1c levels in patients of all groups decreased significantly from baseline. There were significant differences in the proportions of patients that achieved HbA1c <7% between groups, especially in group C and group D, compared with group A at week 24 (60.4, 62.2% vs. 25.5%, all *p* < 0.05). 1,5-Anhydroglucitol changes were obvious in group A and group C at week 24 from baseline (all *p* < 0.05 within groups). Factorial analysis of ANOVA showed that MPA intervention was the main effective factor for HbA1c change (*F* = 4.59, *p* = 0.034), and there was no effect on HbA1c change for SMBG intervention (*p* = 0.975)	Hypoglycemia was the major adverse event in the study No severe hypoglycemia or serious adverse events were reported	The mobile phone–based glucose-monitoring and feedback system was effective in glycemic control when applied in primary care clinic settings. This system could be utilized effectively with diverse institutions and patients. MPA intervention is the main factor for HbA1c change
Brath et al. (50)	T2DM	RCT (single-blinded)	Custom/ unspecified app	53	Adherence: Significant difference in diabetes medication adherence between monitoring and control phases: Minimal adherence was 89% for metformin treatment in the CON: control phase and 93% in the MON: monitoring phase and was above 90% for the other drugs in the CON as well as in the MON phase	None	mHealth-based adherence management is feasible and well accepted by patients with increased cardiovascular risk It may help to increase adherence, even in patients with high baseline adherence and, subsequently, lead to improved therapy control
Yang et al. (51)	T2DM	Cluster-RCT	Hicare smart K app	145	HbA1c level: At 3 months, participants in the intervention group showed significantly more improvement in HbA1c (adjusted mean difference to control −0.30, 95% CI −0.50 to −0.11; *p* = 0.003) and fasting plasma glucose (−17.29 mg/dL, 95% CI −29.33 to −5.26; *p* = 0.005) than those in the control group BP: Reduction in blood pressure	None	Mobile app may not influence adherence-related beliefs in 6 months App may be effective for patients with lower self-efficacy and LOC beliefs. Future research should test the effectiveness of mobile technology with theory-based trials

### 3.2 Validity and rigor

Concerning the validity and rigor of the selected articles, the literature search for the state of the art was intended to provide a global overview of the disease, the condition, the technology used, and to provide a baseline for the performance and safety profile. It was not intended to be extensive from a regulatory perspective. The study relied on the fact that the selected articles came from peer-reviewed journals and have been identified and selected through a well documented protocol.

## 4 Discussion

### 4.1 Principal findings

After the analysis of the extraction table referring to the included 35 primary studies, 24 studies (68.6%) analyzed clinical indications for type 2 diabetes mellitus (T2DM), six studies (17.1%) analyzed clinical indications for cardiovascular conditions, three studies (8.7%) analyzed clinical indications for cancer, one study (2.8%) analyzed clinical indications for chronic obstructive pulmonary disease (COPD), and one study (2.8%) analyzed clinical indications for hypertension. No severe adverse events related to the use of mHealth were reported in any of them. However, five studies (14.3%) reported mild adverse events (related to hypoglycemia, uncontrolled hypertension) and four studies (11.4%) reported technical issues with the devices (related to missing patient adherence requirements, Bluetooth unsuccessful pairing, and poor network connections).

**Figure 6:**
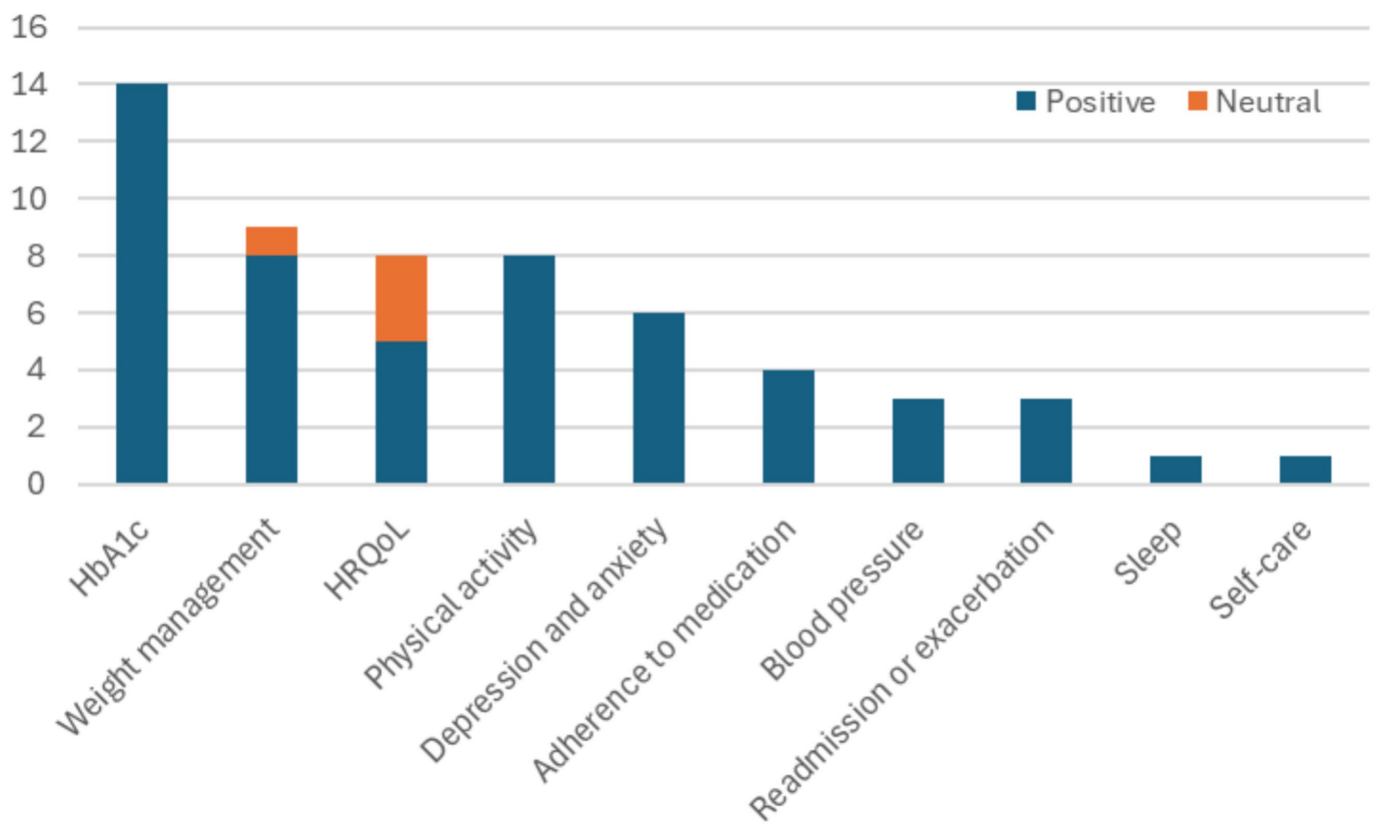
Variables of interest considered across the 35 studies.

Regarding the variables of interest, out of the 35 studies, the observations are summarized in Figure 6 and are also listed below:

- Fourteen studies reported positive results on the reduction of glycated hemoglobin (HbA1c) with the use of mHealth devices.
- Eight studies examined health-related quality of life (HRQoL); in three cases, there were no statistically significant differences, while the groups using mHealth devices in the other five studies experienced better HRQoL.
- Seven studies focused on physical activity and performance, while one study focused on the six-minute walk distance (6MWD), all reflecting increased attention to physical activity levels.
- Six studies addressed depression and anxiety, with mostly self-reported benefits observed.
- Four studies reported improvements in body fat. Three studies addressed BMI, with one finding no statistically significant change and two BMI reduction. Two studies reported significant weight or waist circumference reduction.
- Four studies reported adherence to medications in the mHealth solutions arm.
- Three studies examined blood pressure, noting reductions.
- Two studies reported reduced hospital readmissions, while one reported improvement in exacerbations.
- One study noted improvements in sleep time.
- One study reported improvement in self-care/management.

What can be observed considering the variables of interest is that clinical outcomes are important in mHealth solutions, since HbA1c, blood pressure, readmissions and exacerbations are considered 20 times. On the other hand, there is a definite shift toward behavioral variables. Objectively measured behavioral variables (weight-related, physical activity and sleep) are considered 18 times, while subjectively reported variables (HRQoL, depression, anxiety, adherence to medication and self-care) are considered 19 times.

### 4.2 The desired app characteristics

Based on the principal findings of the systematic literature review, it is apparent that following features and essential characteristics for SaMDs that can support chronic disease patients. We can categorize features in terms of functionalities or technical feature and health outcomes.

#### 4.2.1 Technical features

- **PROMs**: Collecting licensed or custom questionnaires, such as EQ-5D-5L, capture patient experience directly and can give useful information to the healthcare professionals (HCPs) like
  - Enhancing clinical decision-making
  - Improving treatment personalization
  - Facilitating regulatory compliance
  - Demonstrating real-world effectiveness
    The key features that are important when using PROMs are the scheduling that should be based actions or dates orchestrated by clinical pathways and the personalization of PROMs delivery according to patient’s status.
- **Notifications**: This functionality uses the push notifications of the smartphones and communicates to the patients any type of message. Questionnaires can be sent via this functionality, medication reminders as well as any other type of message to remind patients to do certain actions like expiring questionnaires or other tasks like inserting their weight.
  The key features here are to allow flexibility on using them and a key feature is to be able to schedule them automatically as per the needs of a clinical protocol but also allowing the clinician to send them instantly to an individual or a group of patients.
- **Alerts**: An alert dashboard for HCPs in a software as a medical device (SaMD) is very valuable to help prioritizing and management of time to cater for patients that need more immediate attention.
  The key feature for such a tool is to facilitate evidence-based decision-making by presenting key metrics and offer data-driven care. Some other features could be summarized below:
  - Real-time monitoring: Enables quick identification of critical issues or changes in patient conditions.
  - Efficiency: Streamlines workflow by consolidating important information in one place.
  - Early intervention: Allows for timely medical responses, potentially preventing complications.
  - Compliance tracking: Assists in monitoring adherence to treatment plans or medication schedules.
- **Messaging and teleconsultation**: These features establish a communication channel between the HCPs and the patients via messages inbox integrated with teleconsultation provision. Some key benefits are the following:
  - Reduction of burden: Both clinicians and patients can benefit from a virtual visit instead of a physical one in hospital saving time and resources.
  - Efficiency: Clinician’s burden is reduced as a lot of messages can be dealt with by the nurses.
  - Early intervention: It allows for timely medical responses, potentially preventing complications.
  - Enhanced patient engagement: Patients report if anything seems off or if they have a question concerning their treatment plan.
- **Conversational agent**: This feature is also called chatbot or agent. The functionality is to give assistance to the patient once they have asked for help on predefined topics. It can be patient or system initiated, starting a dialog, and offering predefined topics and solutions to the user.
  The key features for such agents are to be also system initiated based on certain parameters and it can be used to offer personalized behavioral change coaching through the interaction with the patient based on their progress.
- **Goal setting**: This feature is great to enhance patient engagement and track progress toward a health goal. Here are some key features:
  - Personalized goal creation: Allow patients and healthcare providers to collaboratively set tailored goals.
  - Progress tracking: Visual representations of goal progress over time.
  - Reminders and notifications: Automated alerts to keep patients engaged with their goals.
  - Adjustable targets: Flexibility to modify goals based on patient progress or changing circumstances.
  - System feedback: Recognition of wins and achievements to motivate.
  - Smart goals: AI models to predict a patient’s individual trajectory based on assessing their starting point.

#### 4.2.2 Health outcomes

- **Symptom/condition tracking**: Further to the PROMs, one question widgets or widgets can be allocated to capture mood, symptoms, pain scores, and so on.
- **Medication management**: This feature is very helpful to both clinicians and patients. The benefit for the clinician is to monitor medication adherence from a dashboard after setting up and managing their medication plan with history of drugs, dosages, and frequencies. The patients, on the other hand, will see their medication on their phone and can set up to receive medication reminders or not in order to confirm that the medication was taken.
- **Measurements**: To offer a closer monitoring of patients, some require the use of medical devices to capture daily or, in other frequencies, vitals like spO_2_, blood pressure, weight, blood glucose, and others. This can be dealt with a manual logging of data of via integration with digital devices. These data points are transferred to the platform and can be viewed by clinicians. A key feature would be to set up rules in order to alert when a critical issue is identified or there is a change in trend of a major value, indicating a change in a patient’s condition.
- **Lab exams:** Similar to the measurements, values of biomarkers can be inserted manually or through an integration with a medical device and can be viewed in the platform for clinical decision.
- **Activity tracking**: The literature is very clear on the importance of physical activity in terms of steps and activity minutes mainly, but also resulting information regarding the kilocalories (kcal) spent on such activity. This information helps the clinicians assess the patient health status. The reduction in steps is a very clear deterioration of health if it is accompanied by good treatment adherence and other symptoms. The key feature here is the integration with activity trackers in order to also get the variety of data points that can be obtained from the wearable.
- **Sleep**: Sleep tracking is an important feature for many health-related SaMDs. The key aspects to take into consideration when tracking sleep are duration, time of sleep onset, and quality metrics related to rapid eye movement (REM) and time awake. To receive such information, it is key to have a wearable integration. Other key features are as follows:
  - Sleep pattern visualization: Provide graphs or charts showing sleep trends over time.
  - Sleep hygiene recommendations: Offer personalized tips based on tracked data.
  - Sleep goal setting: Enable users to set and track sleep-related goals.
  - Environmental factors: Log room temperature, noise levels, or light exposure that may affect sleep.
  - Correlation with other health data: Show relationships between sleep and other tracked health metrics.
  - Sleep score: Provide a simplified daily score to help users quickly gage their sleep quality.
  - Reminders: Send notifications for consistent bedtimes or pre-sleep routines.
- **Nutrition**: Last but not least, it is very common when discussing chronic diseases to discuss nutritional habits and consumption of nutrients. Incorporating nutrition tracking and management into an SaMD can be highly beneficial for patient health. The key features for a nutrition component are as follows:
- **Food logging**: Allow users to record meals and snacks, with a searchable database that can be a challenge when only local products are available.
- **Nutrient tracking**: Monitor intake of calories, macronutrients, and micronutrients.
- **Personalized recommendations**: Offer dietary suggestions based on health goals and conditions.
- **Integration with health data**: Connect nutrition info with other health metrics like blood sugar or weight.
- **Visual summaries**: Display charts of nutritional intake over time.
- **Goal setting**: Allow users to set and track nutrition-related goals.
- Educational content: Provide information on balanced diets and nutrition basics.
- **Recipe suggestions**: Offer healthy recipe ideas aligned with dietary needs.
- **Hydration tracking**: Include water intake monitoring.

### 4.3 Conclusion

The systematic literature review demonstrates the significant potential of SaMD and mHealth applications in revolutionizing chronic disease management through remote patient monitoring (RPM) and digital therapeutics (DTx). The evidence synthesized from multiple systematic reviews and clinical studies indicates that these technologies, exemplified by solutions like Healthentia, can effectively support patient monitoring and improve health outcomes while meeting crucial safety and performance requirements. The positive results observed across various chronic conditions underscore the transformative role of digital health interventions in modern healthcare delivery. However, further research is needed to address long-term efficacy, cost-effectiveness, and integration into existing healthcare systems. As the field rapidly evolves, continued evaluation and refinement of these technologies will be essential to fully realize their potential in enhancing patient care and health management strategies.

### 4.4 Future research

The health outcomes presented in the principal findings are mainly connected to the increased adherence of the patients. It is apparent that SaMDs that offer high levels of engagement with the patient can increase adherence and result in positive health outcomes. However, the impact of lifestyle change, which is the common denominator in chronic diseases, is not considered. The future research will be focused on the benefits of virtual coaching and how this can consume all data captured by the device and processed in a way to orchestrate behavioral change techniques with a strong impact on the patient’s health.

The growing adoption of RPM signifies a shift toward more patient-centered, data-driven healthcare delivery. Looking ahead, the integration of advanced technologies like artificial intelligence and machine learning into RPM platforms is expected to further optimize care delivery and decision-making processes.

## Data Availability

The raw data supporting the conclusions of this article are available through the cited papers, as it is a systematic literature review.
